# Fenofibrate Ameliorates Hepatic Ischemia/Reperfusion Injury in Mice: Involvements of Apoptosis, Autophagy, and PPAR-*α* Activation

**DOI:** 10.1155/2021/6658944

**Published:** 2021-02-01

**Authors:** Jie Zhang, Ping Cheng, Weiqi Dai, Jie Ji, Liwei Wu, Jiao Feng, Jianye Wu, Qiang Yu, Jingjing Li, Chuanyong Guo

**Affiliations:** ^1^Department of Gastroenterology, Shanghai Tenth People's Hospital, Tongji University School of Medicine, Shanghai 200072, China; ^2^Shanghai Tenth Hospital, School of Clinical Medicine of Nanjing Medical University, Shanghai 200072, China; ^3^Department of Gerontology, Shanghai Minhang District Central Hospital, Shanghai 201100, China; ^4^Department of Gastroenterology, Putuo People's Hospital, Tongji University School of Medicine, Shanghai 200060, China

## Abstract

Hepatic ischemia and reperfusion injury is characterized by hepatocyte apoptosis, impaired autophagy, and oxidative stress. Fenofibrate, a commonly used antilipidemic drug, has been verified to exert hepatic protective effects in other cells and animal models. The purpose of this study was to identify the function of fenofibrate on mouse hepatic IR injury and discuss the possible mechanisms. A segmental (70%) hepatic warm ischemia model was established in Balb/c mice. Serum and liver tissue samples were collected for detecting pathological changes at 2, 8, and 24 h after reperfusion, while fenofibrate (50 mg/kg, 100 mg/kg) was injected intraperitoneally 1 hour prior to surgery. Compared to the IR group, pretreatment of FF could reduce the inflammatory response and inhibit apoptosis and autophagy. Furthermore, fenofibrate can activate PPAR-*α*, which is associated with the phosphorylation of AMPK.

## 1. Introduction

Ischemia/reperfusion (I/R) injury is a major concern during surgical procedures such as liver resection, trauma, and transplantation, which can lead to liver injury or even failure for its inevitable interruption and subsequent restoration of circulation [1, 2]. Hepatic IR is a complex phenomenon, and its various mechanisms have been investigated extensively. As the main target, hepatocytes are attacked by hypoxia, nutrient deprivation, and oxidative stress in ischemia/reperfusion injury [3]. IR-resulted hepatocytes then produce damage-associated molecular patterns (DAMPs), which trigger immune responses and inflammation [4]. Proinflammatory cytokine-mediated apoptosis and reactive oxygen species- (ROS-) induced necrosis is the leading cause of hepatocyte death in IRI [5]. Autophagy is an intracellular self-digestive lysosomal recycling pathway, and its role in hepatic IR injury has been widely studied [4, 6].

Fenofibrate belongs to the group of fibrate drugs, which are generally used in the treatment of dyslipidemia and combined hyperlipidemia patients [7]. Also, fenofibrate is known as peroxisome proliferator-activated receptor-*α* (PPAR*α*) agonist, the nuclear receptor superfamily member, which was discovered in 1990 [8]. As an activator of nuclear receptor, fenofibrate regulates gene/protein interactions that are involved in various pathophysiological processes, such as regulation of *β*-oxidation of fatty acids, inflammation, oxidative stress, and even tumorigenesis and cancer progression [9, 10]. Several studies have shown anti-inflammatory, antioxidant, and antiapoptotic effects of fenofibrate to attenuate I/R injury in the brain, heart, kidney, and intestine [11–14]. But to date, no literatures have been reported for the liver protection of fenofibrate in hepatic I/R injury of mice. Moreover, the specific mechanisms of fenofibrate in IR remain unclear.

AMPK, adenosine monophosphate–activated protein kinase, a key sensor of cellular nutrient supply and energy status, plays crucial roles in regulating cellular growth and metabolism and is related to processes such as autophagy in eukaryotes [15]. It was observed that the activation of the AMPK pathway inhibited macrophage activation to prevent inflammation response [16]. Fenofibrate has a therapeutic effect on HFD-induced kidney injury, through the activation of AMPK and induction of subsequent downstream effectors: autophagy and antioxidants [17]. Experiment demonstrated that fenofibrate activates AMPK in endothelial cells, leading to reduce inflammation, as well as inhibition of apoptosis [18]. The activation of AMPK is a trigger to downstream mediators, including PPAR-*α*, and they together participate in mechanisms like inflammation, apoptosis, autophagy, and oxidative stress that are involved in IR. There are reasons to believe fenofibrate may function through activating the AMPK/PPAR-*α* pathway.

Herein, we evaluated the value of fenofibrate in hepatocellular protection during hepatic ischemia/reperfusion injury. The present study investigated whether and how fenofibrate administration would affect liver functions and its underlying mechanisms in a well-established murine IR model based on our previous research [19]. We hypothesized that FF could function by inhibiting inflammation response, apoptosis, and autophagy in a PPAR-*α*- and AMPK-dependent manner.

## 2. Materials and Methods

### 2.1. Reagents

Fenofibrate was purchased from Kingmorn Life Science (Shanghai, China) and suspended with 10% DMSO. ALT and AST regent kits were obtained from Jiancheng Bioengineering Institute (Nanjing, China). The enzyme-linked immunosorbent assay (ELISA) kits were acquired from eBioscience (San Diego, CA, USA). The RNA polymerase chain reaction (PCR) kit was from Takara Biotechnology (Dalian, China). The antibodies for PPAR-*α*, IL-6, Bax, Bcl-2, Beclin-1, P62, LC3, Casepase-9, Caspase-3, and Nrf-2 were provided by Proteintech (Chicago, IL, USA). The TNF-α and IL-1*β* antibodies were from Abcam (Cambridge, MA, USA). The AMPK and p-AMPK antibodies were from CST (Danvers, MA, USA). The TdT-mediated dUTP nick end labeling (TUNEL) apoptosis assay kit was from Roche (Roche Ltd., Basel, Switzerland).

### 2.2. Animals

Male Balb/c mice weighing 23 ± 2 g and aged 6–8 weeks old were obtained from Shanghai SLAC Laboratory Animal Co. Ltd. (Shanghai, China). The mice were group-housed in standard plastic cages at ambient temperature (23 ± 1°C) and 55% humidity with a 12 h light-dark cycle, having access to food and water ad libitum. All animal experiments were carried out in accordance with the National Institutes of Health Guidelines and with approval of the Animal Care and Use Committee of Shanghai Tongji University.

### 2.3. Treatment Protocol

78 mice were randomly assigned to one of five groups as follows: (a) normal control group (*n* = 6); (b) sham group (*n* = 18); (c) I/R without any pretreatment (*n* = 18); (d) I/R with pretreatment of FF (50 mg/kg); and (e) I/R with pretreatment of FF (100 mg/kg). Fenofibrate was administered by intravenous route 1 h before surgery. A total of six mice were selected to sacrifice from each group (except normal control group) at 2, 8, and 24 hours after reperfusion to obtain blood and tissue samples.

Blood samples were collected and then placed at 4°C for 5 hours. Serum was separated by centrifugation of the blood at 4600 × g (4°C) for 10 minutes. Serum was collected, aliquoted, and stored at −80°C until the biochemical analysis. A portion of the median and left liver lobes was quickly isolated and preserved in 4% paraformaldehyde solution for at least 24 hours at 4°C for histopathological assessment while the remaining liver tissue was collected, snap frozen with liquid nitrogen, and stored at −80°C for the subsequent experiments.

### 2.4. I/R Model Establishment

A model of segmental (70%) hepatic warm IR was used, as previously described [6]. Food was withheld for a period of 12 h before surgery, but mice had free access to water. Mice were anesthetized with 1.25% sodium pentobarbital (Nembutal, St. Louis, MO, USA) by injection intraperitoneally, and then a midline laparotomy was performed.

The hepatoduodenal ligament was dissected, and a microvascular atraumatic clamp was placed on the portal pedicle to the median and left lobe of the liver for partial hepatic ischemia. After 45 minutes, the clamp was removed to initiate liver reperfusion and the wound was sewn with 4–0 silk. Sham groups were subjected to the same procedure but without vascular occlusion. A constant warm body temperature was needed to be maintained during the procedure.

### 2.5. Determination of Serum Parameters

Serum alanine aminotransferase (ALT) and aspartate aminotransferase (AST) were measured with commercially available colorimetric assay kits as described by the manufacturers of Jiancheng Bioengineering Institute. The amount of IL-1*β* and TNF-*α* was determined using ELISA kits, following the manufacturer's instructions.

### 2.6. Histopathological Evaluation

The prepared liver specimens were dehydrated with ethanol and embedded in paraffin. Samples were sliced into 5 *μ*m thick sections and stained with hematoxylin and eosin (H&E). Inflammation and tissue damage were confirmed with a light microscope.

### 2.7. Western Blot Analysis

Total protein of liver tissue was extracted from liver tissues stored in liquid nitrogen with radioimmunoprecipitation assay (RIPA) lysis buffer mixed with protease inhibitors (PI) and phenylmethyl-sulfonyl fluoride (PMSF). Protein concentration was quantified using the bicinchoninic acid protein assay kit (Kaiji, China). Equal amounts of total protein were separated on 7.5–12.5% SDS-polyacrylamide gels and then transferred onto 0.22 *μ*m polyvinylidene fluoride membranes. Nonspecific binding sites were blocked with PBS containing 5% nonfat milk for at least 1 h at room temperature, and the membranes were incubated overnight at 4°C with the following primary antibodies and dilutions: *β*-actin (1 : 1,000), TNF-*α* (1 : 1,000), IL-1*β* (1 : 1,000), IL-6 (1 : 1,000), LC3 (1 : 500), Beclin-1 (1 : 500), Bcl-2 (1 : 1,000), Bax (1 : 1,000), caspase3 (1 : 1,000), caspase9 (1 : 1,000), P62 (1 : 1,000), PPAR-*α* (1 : 500), AMPK (1 : 1,000), p-AMPK (1 : 1,000), and Nrf-2 (1 : 1,000). The next day, membranes were washed three times for 10 min each using PBST (PBS containing 0.1% Tween 20) and then incubated with secondary antibodies at 1 : 2000 for 1 h at room temperature protected from light. Membranes were then washed 3 times with PBST and scanned with the Odyssey two-color infrared laser imaging system (LI-COR, Lincoln, NE, USA). The gray values were quantified using ImageJ analysis software.

### 2.8. Immunohistochemistry

Paraffin-embedded liver sections were dewaxed in xylene and dehydrated with gradient alcohol. Antigen retrieval was performed by citrate buffer and incubated in a 95°C water bath for 20 min. To block endogenous peroxidases, the sections were incubated with 3% hydrogen peroxide for 10 min at room temperature. Sections were washed with PBS three times and then treated with 5% bovine serum albumin (BSA) for 20 min to block nonspecific proteins. Next, the liver specimens were incubated overnight at 4°C with the following primary antibodies and dilutions: anti-TNF-*α*, anti-IL-1*β*, anti-Bcl-2, anti-Bax, anti-Beclin-1, anti-PPAR-*α*, and anti-Nrf-2 (all 1 : 200), anti-LC3, and p-AMPK (1 : 100), followed by incubation in secondary antibody (1: 50) for 1 h at 37°C. A diaminobenzidine (DAB) kit was used to analyze antibody binding under a light microscope. The stained area was measured by using Image-Pro Plus software (version 6.0).

### 2.9. RNA Isolation and Quantitative Real-Time PCR (RT-qPCR)

Total RNA was extracted from stored frozen liver specimens using TRIzol reagent (Tiangen Biotech, China). RNA was reverse-transcribed into cDNA with a Reverse-Transcription Kit (TaKaRa Biotechnology, China). Gene expression was measured using SYBR Premix Ex Taq (TaKaRa Biotechnology, China), and cDNA was quantified with the 7900HT Fast Real-Time PCR System (Applied Biosystems, CA, USA). Oligonucleotide primer sequences are listed in Table 1. The relative expression levels were analyzed using the 2−△△Ct method and normalized relation to *β*-actin.

### 2.10. TUNEL Staining

The hepatocyte apoptosis was determined by TUNEL assay. Followed by dewaxing and rehydrating, the prepared 5 *μ*m sections were then digested with 20 *μ*g/ml proteinase K for 30 min. After washing 4 times with PBS, the sections were added with TUNEL reaction mixture. Finally, the positive areas were observed by the light microscope.

### 2.11. Statistical Analysis

Experimental data were presented as the mean ± standard deviation (SD), and experiments were repeated at least three times. Statistical differences between groups were analyzed by Student's *t*-test and one-way analysis of variance (ANOVA) followed by Tukey's post hoc test. *P* < 0.05 was considered statistically significant. Statistical analyses were performed, and the graphic charts were plotted by GraphPad Prism 6 software.

## 3. Results

### 3.1. FF Improved Hepatic Structure and Function of Mice Subjected to IR Injury

**Figure 1:**
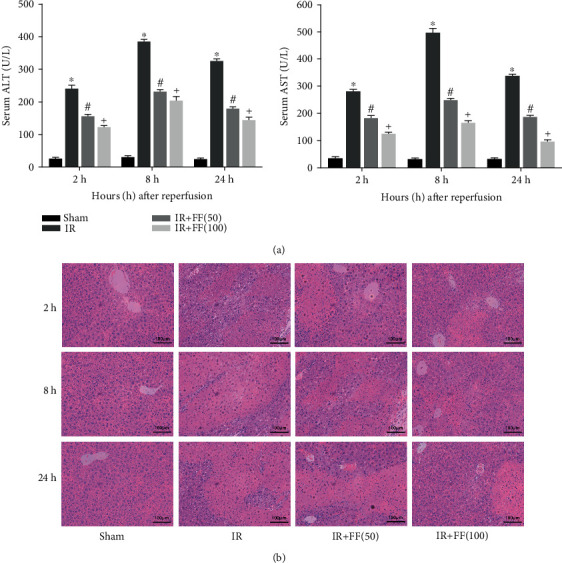
Fenofibrate pretreatment ameliorated hepatic function in IR-induced liver injury. (a) The levels of serum ALT and AST are presented as mean ± SD (*n* = 6; ^∗^*P* < 0.05 for IR vs. sham; ^#^*P* < 0.05 for IR+FF (50) vs. IR; ^+^*P* < 0.05 for IR+FF (100) vs. IR; ^*P* < 0.05 for IR+FF (50) vs. IR+FF (100)). (b) Liver sections were stained with H&E and examined under light microscopy (magnification, 200x).

The levels of biomarkers of hepatic function, ALT and AST, in serum were significantly elevated in the IR group at 3 time points (Figure 1(a)). This indicated the successful establishment of a segmental hepatic IR model. In parallel, we observed the aminotransferase was at its highest level at 8 h. ALT and AST at the same time points were markedly lower in the fenofibrate-treated groups. The variation, which was more pronounced in the high-dose compared with the low-dose group. To further determine the drug effect on HIRI, we performed H&E staining. The pathological changes showed that in the sham group, liver tissue structure remained intact, whereas marked congestion, edema, necrosis, massive neutrophil infiltration, and accumulation appeared in the IR group at the three time points (especially in the 24 h group) (Figure 1(b)). Pretreatment with FF at 50 and 100 mg/kg alleviated the liver histopathological alterations in IR groups. From the above results, it could be concluded that fenofibrate exerted a protective effect on liver injury, and the higher the dose, the better the effect.

### 3.2. FF Prevented Hepatic and Systemic Inflammation Induced by Hepatic IR

**Figure 2:**
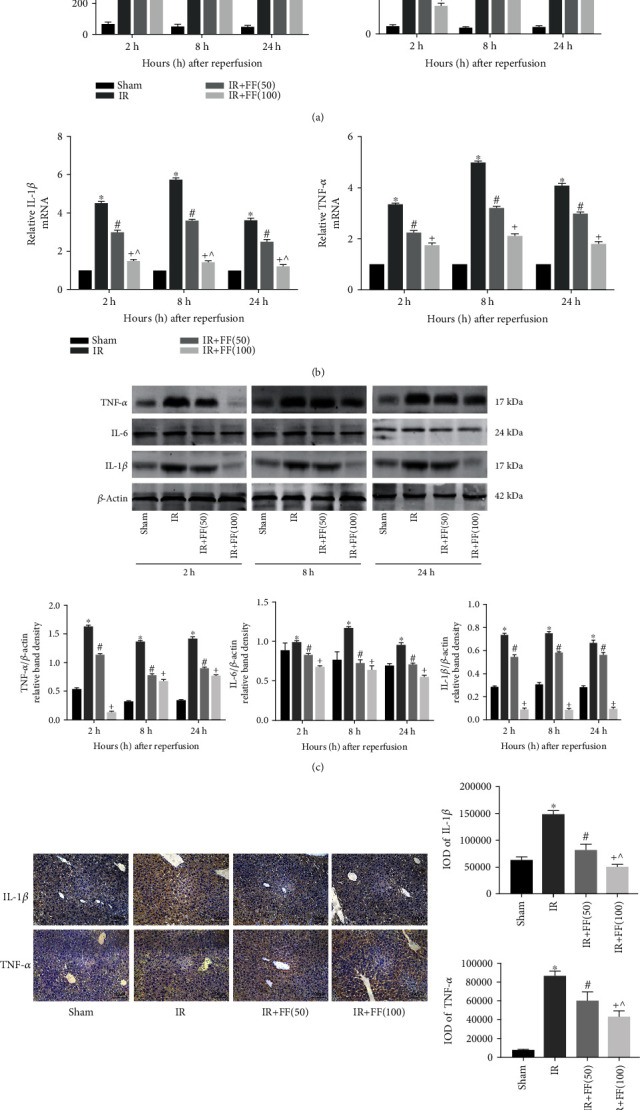
Fenofibrate reduced the expression of inflammatory cytokines. (a) Serum IL-1*β* and TNF-*α* were detected by ELISA. (b) Relative IL-1*β* and TNF-*α* mRNA levels were determined by qRT-PCR. (c) Western blot analysis of TNF-*α*, IL-6, and IL-1*β* protein levels. The western blot results were quantified with ImageJ 8.0 software. (d) Immunohistochemical staining (200x) showed expression of IL-1*β* and TNF-*α* protein in liver tissues at 8 hours after reperfusion. Final evaluations were made using Image-Pro Plus 6.0 software to calculate the IOD of the positive staining area. Data are presented as mean ± SD (*n* = 6; ^∗^*P* < 0.05 for IR vs. sham; ^#^*P* < 0.05 for IR+FF (50) vs. IR; ^+^*P* < 0.05 for IR+FF (100) vs. IR; ^*P* < 0.05 for IR+FF (50) vs. IR+FF (100)).

It has been suggested that the release of inflammatory factors strongly promotes HIR injury. So, TNF-*α*, IL-6, and IL-1*β*, the proinflammatory cytokines, were detected to explore the effect of FF on inflammation in terms of serology, protein levels, and gene transcription. As shown by ELISA results, the expression of TNF-*α* and IL-1*β* in serum was significantly higher than that in the sham group and peaked at 8 hours after reperfusion (Figure 2(a)). The protein levels also increased in the IR group as shown by western blotting (Figure 2(c)). In addition, the results of immunohistochemistry (IHC) staining and the mRNA expression of PCR were consistent with the results mentioned above (Figures 2(b) and 2(d)). Pretreatment of mice with fenofibrate significantly diminished the expression of inflammatory factors subjected to IR at all time points, and this effect was evident at 100 mg/kg dose. Besides, in H&E staining, the IR group exhibited more inflammatory cell infiltration than the other groups (Figure 1(b)). In summary, inflammatory cascades induced by hepatic ischemia/reperfusion injury in mice could be inhibited by fenofibrate.

### 3.3. FF Improved Apoptosis Induced by HIR

**Figure 3:**
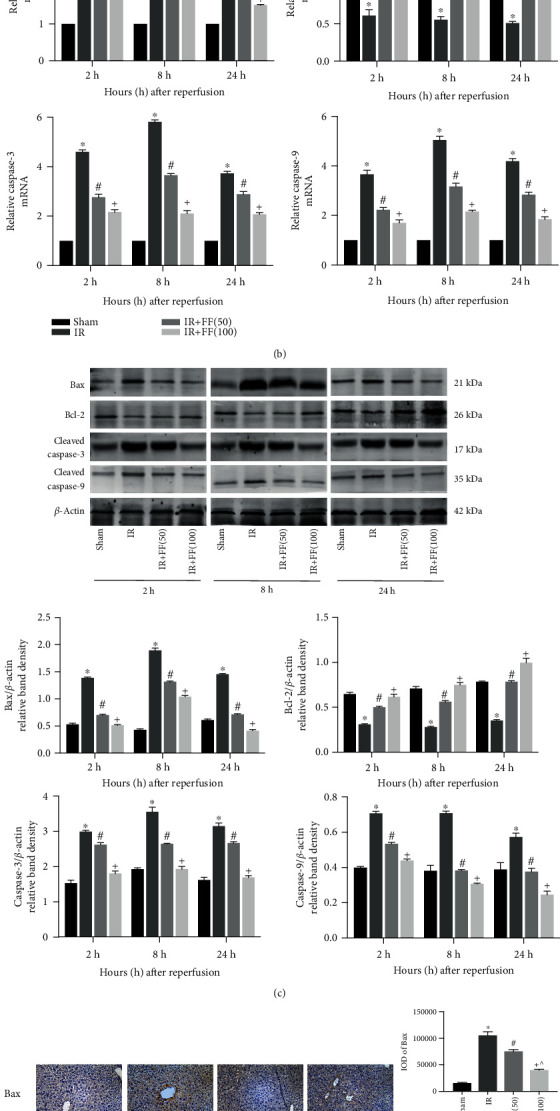
Fenofibrate attenuated IR-induced apoptosis. (a) After 8 h reperfusion, liver tissues were stained by TUNEL and observed under microscopy (magnification, 200x). Final evaluations were made using Image-Pro Plus 6.0 software to calculate the percentage of positive cells. (b) Relative Bax, Bcl-2, Caspase-3, and Caspase-9 mRNA levels were determined by qRT-PCR. (c) Western blot analysis of Bax, Bcl-2, cleaved Caspase-3, and cleaved Caspase-9 levels. The western blot results were quantified with ImageJ 8.0 software. (d) Bax and Bcl-2 protein expressions in liver tissues at 8 hours after reperfusion are shown by immunohistochemical staining (200x). Final evaluations were made using Image-Pro Plus 6.0 software to calculate the IOD of the positive staining area. Data are presented as mean ± SD (*n* = 6; ^∗^*P* < 0.05 for IR vs. sham; ^#^*P* < 0.05 for IR+FF (50) vs. IR; ^+^*P* < 0.05 for IR+FF (100) vs. IR; ^*P* < 0.05 for IR+FF (50) vs. IR+FF (100)) (reproduced from Ji et al. [19]).

Large area of hepatocyte apoptosis is one of the serious consequences of hepatic IR. Therefore, alleviated apoptosis becomes an essential part of IRI treatment. First of all, TUNEL staining assay was used to evaluate the degree of apoptotic death from samples at 8 hours after reperfusion. The results indicated that apoptotic hepatocytes were largely observed in tissues of the IR group, while TUNEL-positive cells were significantly decreased in the fenofibrate pretreatment group (Figure 3(a)). Performing qRT-PCR, western blotting, and IHC to detect the expression of apoptosis-related markers, we found that the mRNA and protein expressions of Bax, Caspase-3, and Caspase-9 were elevated in the IR group and downregulated in IR+FF (50 mg/kg and 100 mg/kg) groups at each time points after reperfusion. Meanwhile, Bcl-2, an antiapoptotic protein, showed a marked drop in the IR group but was obviously upregulated in fenofibrate preconditioning groups (Figures 3(b) and 3(c)). The expression of Bax and Bcl-2 on IHC staining, distinctly shown in Figure 3(d), presented a similar trend as on PCR and western blotting. In conclusion, fenofibrate can also reduce apoptosis in IR-induced liver injury.

### 3.4. FF Inhibited Hepatocyte Autophagy

**Figure 4:**
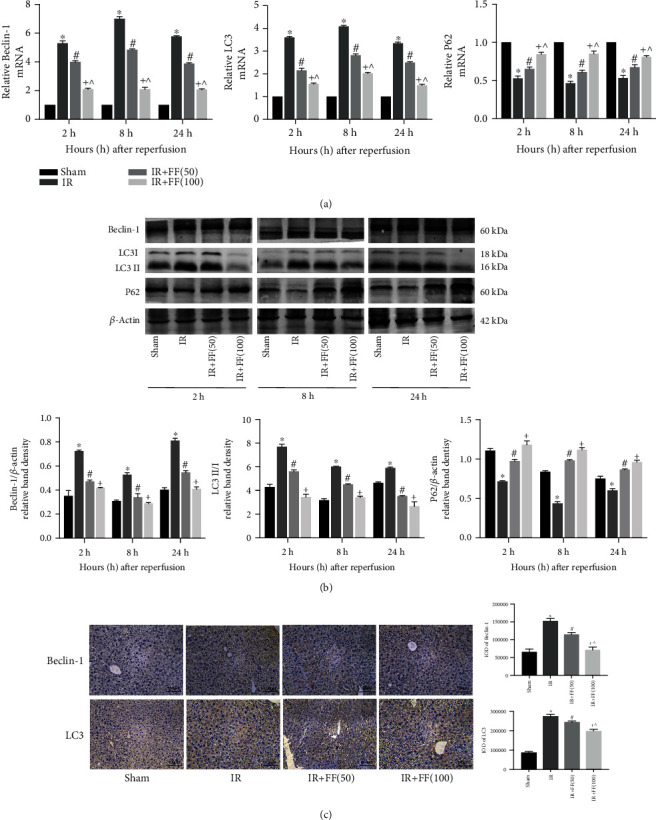
Fenofibrate inhibited autophagy activity during IR injury. (a) Relative Beclin-1, LC3, and P62 mRNA levels were determined by qRT-PCR. (b) Western blot analysis of Beclin-1, LC3, and P62 protein levels. The western blot results were quantified with ImageJ 8.0 software. (c) Beclin-1 and LC3 protein expression in liver tissues at 8 hours after reperfusion was shown by immunohistochemical staining (200x). Final evaluations were made using Image-Pro Plus 6.0 software to calculate the IOD of the positive staining area. Data are presented as mean ± SD (*n* = 6; ^∗^*P* < 0.05 for IR vs. sham; ^#^*P* < 0.05 for IR+FF (50) vs. IR; ^+^*P* < 0.05 for IR+FF (100) vs. IR; ^*P* < 0.05 for IR+FF (50) vs. IR+FF (100)) (reproduced from Ji et al. [19]).

It is well known that autophagy plays a vital role in hepatic IR injury. Autophagy-associated markers including LC3, Beclin-1, and P62 were evaluated to further assess the potential role of FF in IR injury. The expression of Beclin-1 and LC3 in hepatic tissues was detected by real-time PCR. The results revealed that hepatic IR obviously activated the transcription of Beclin-1 and LC3 compared to the sham group. The expression of antiautophagy protein P62 presented an opposite trend to LC3 and Beclin-1. When FF was taken to the mice, Beclin-1 and LC3 expressions were downregulated and P62 expressions were upregulated. (Figure 4(a)). Western blotting results were in accordance with this trend (Figure 4(b)). Analyses of IHC further confirmed the antiautophagy effect of FF (Figure 4(c)). Thus, we deduced from our results that FF could inhibit autophagy in a dose-dependent manner during hepatic IR injury in mice.

### 3.5. FF Attenuated the Downregulation of the PPAR-*α* during HIR

**Figure 5:**
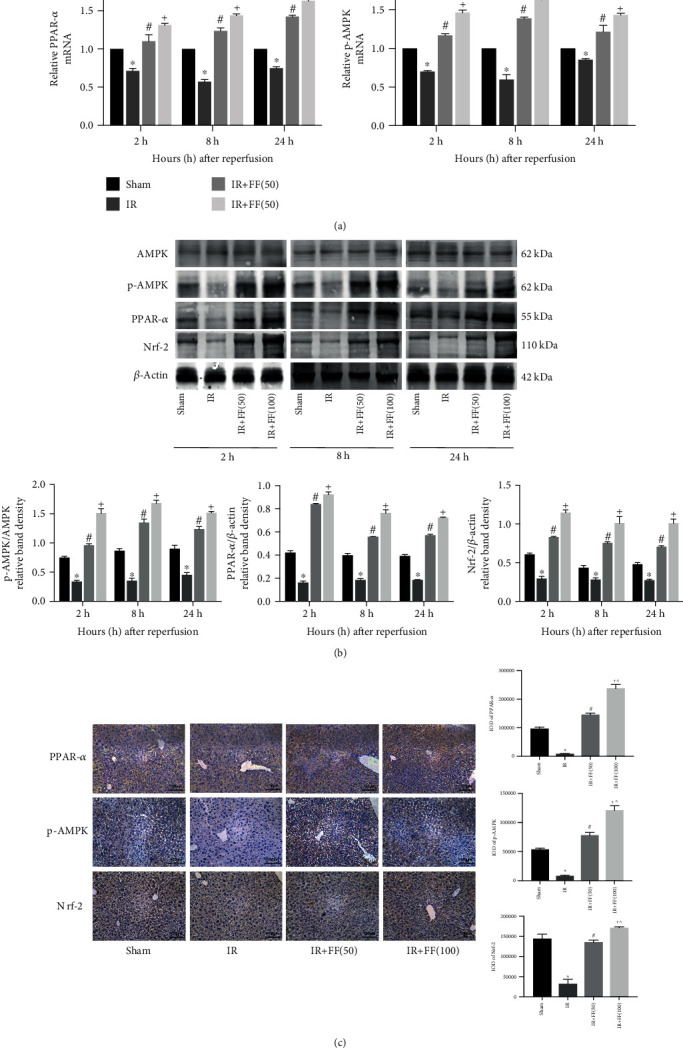
The protective effect of fenofibrate during hepatic IR injury is closely related with the activation of PPAR-*α*. (a) Relative PPAR-*α* and p-AMPK mRNA levels were determined by qRT-PCR. (b) Western blot analysis of AMPK, p-AMPK, PPAR-*α*, and Nrf-2 levels. The western blot results were quantified with ImageJ 8.0 software. (c) Levels of p-AMPK, PPAR-*α*, and Nrf-2 in liver tissues at 8 hours after reperfusion are shown by immunohistochemical staining (200x). Final evaluations were made using Image-Pro Plus 6.0 software to calculate the IOD of the positive staining area. Data are presented as mean ± SD (*n* = 6; ^∗^*P* < 0.05 for IR vs. sham; ^#^*P* < 0.05 for IR+FF (50) vs. IR; ^+^*P* < 0.05 for IR+FF (100) vs. IR; ^*P* < 0.05 for IR+FF (50) vs. IR+FF (100).

Fenofibrate, as a PPAR*α* agonist, showed potential hepatoprotective effects on IR from the above results. To explore the underlying mechanism of fenofibrate, we measured PPAR-*α* levels in the liver. The results suggested that PPAR-*α* levels were downregulated in IR groups, and fenofibrate preconditioning could markedly increase the PPAR-*α* expression, consistent with the expression of Bcl-2 and P62. And the high-dose group (100 mg/kg) performed obviously (Figures 5(a)–5(c)).

### 3.6. FF Activated the Phosphorylation of AMPK and the Expression of Nrf-2

To verify our hypothesis, we further assessed the AMPK expression conditions. We could see that fenofibrate did not affect the total expression of AMPK between the four groups. However, phosphorylated AMPK (p-AMPK) showed a clear difference between the IR group and IR+FF (50 mg/kg, 100 mg/kg) groups, which may indicate that fenofibrate could activate the phosphorylation of AMPK. These conclusions could be confirmed by PCR, western blotting, and IHC (Figures 5(a)–5(c)). As it is known that the oxidative stress damage is also one of the major drivers of IR injury, we detected the protein Nrf-2, a nuclear transcription factor associated with antioxidant effect, and found that fenofibrate preconditioning increased the expression and accumulation of Nrf-2 (Figures 5(b) and 5(c)). In conclusion, fenofibrate could take effect by activating the phosphorylation of AMPK and increasing Nrf-2 expression.

## 4. Discussion

Hepatic IR injury is a clinically relevant processes that occurs in liver resection, trauma, and transplantation, which is the paradoxical damage increasing upon reperfusion of ischemic organs [20]. Hence, the underlying mechanisms need to be explored and potential strategies offered for the liver I/R prophylaxis and treatment should be suggested. Fenofibrate, as a known agonist of PPAR alpha, has been commonly used as a clinical drug to modify blood lipids for treatment of hypertriglyceridemia, hyperlipidemia, and cholestatic liver disease like primary biliary cirrhosis [21]. Fenofibrate is verified to ameliorate liver injury such as sunitinib-induced liver damage and concanavalin- or diet-induced hepatitis [22, 23]. It exhibits potential anti-inflammatory, antioxidant, and antiapoptotic properties. In the current study, we would like to confirm the role of fenofibrate on hepatic I/R in mice and explore the mechanisms behind.

For the above purpose, we used a well-established Balb/c mice model of hepatic IR injury. Ischemia-reperfusion process resulted in elevated serum ALT and AST levels, both of which are indicators of early acute hepatic damage, while fenofibrate pretreatment decreased liver enzyme activities, and this protective effect was dose-related. These results were further validated by the findings of pathological changes. The necrotic area and massive inflammatory cell infiltration indicated that FF could reduce the severity of liver injury caused by hepatic IR.

The physiological and pathophysiological processes involved in hepatic IR injury are complicated. During the initial phase, the oxidative phosphorylation levels of hepatocytes decrease due to oxygen deficiency, thus affecting the generation of adenosine triphosphate (ATP). The levels of mitochondrial reactive oxygen species (ROS) production in hepatocytes are out of control under abnormal circumstances such as ATP depletion [2]. Kupffer cells in the liver are activated by ROS, to further induce the release of proinflammatory cytokines, such as IL-6, IL-1*β*, and TNF-*α* [24]. Neutrophils and T cells accumulate and are activated by the Kupffer cells, then stimulate more inflammatory factors, which exacerbate ischemic injury [25]. Besides, proinflammatory cytokines in turn drive the generation of ROS, forming a vicious cycle [4]. In the present study, we first analyzed the expression of inflammatory cytokines in serum and liver tissues by ELISA, western blotting, qRT-PCR, and IHC. Our findings clearly showed that fenofibrate administration attenuated hepatic IR-induced release of TNF-*α*, IL-6, and IL-1*β*. And the favorable anti-inflammatory profiles are potentiated in the high-dose group (100 mg/kg).

The proinflammatory cytokine TNF-*α* plays a pivotal role in various signaling pathways according to previous studies, which could result in extrinsic hepatocyte apoptosis in HIR injury [26]. Moreover, ROS resulting in mitochondrial permeability transition (MPT) can lead to the intrinsic apoptosis pathway. Apoptosis is involved in hepatic IRI [27]. The proapoptotic protein Bax is mostly present in the cytoplasm but migrates to the outer mitochondrial membrane under stimulation, which can lead to the release of intermembrane proteins cytochrome C (Cyto C) to initiate apoptosis. Then, Cyto C subsequently promotes caspase activation and elicits cell death via the intrinsic mitochondrial apoptotic pathway [28]. Bcl-2, the antiapoptosis protein, localized to intracellular mitochondria membranes can restrain the release of cytochrome C during apoptosis to play a protective role [29]. In our experiments, fenofibrate attenuating apoptosis was verified by the results of TUNEL. Next, we detected the expression of protein in connection with apoptosis, including Bcl-2, Bax, Caspase-3, and Caspase-9, to ensure how fenofibrate functioned to reduce the damage of HIRI. As expected, we further found the decreased levels of Bax while increased Bcl-2 in FF-administration groups. Caspase-9 and Caspase-3 had similar trends to those observed for Bax. The above results indicated FF could alleviate hepatocyte apoptosis induced by hepatic IR injury.

There are profound interactions between autophagy and apoptosis: they can mutually reinforce and inhibit in many physical activities [4, 30]. Bcl-2 is the intermediary between apoptosis and autophagy, which is participated in the formation of Beclin-1/Bcl-2 complex. Bcl-2 protein binds to Beclin-1 through its BH3 domain [31]. Under the circumstance of apoptosis, Bcl-2 is inactivated and the complex is divided [32]. Subsequently, the free Beclin-1 promotes the induction of autophagy. Increased Bcl-2 could combine with free Beclin-1 and decrease the conversion of LC3 I to LC3 II, which further blocked autophagosome formation [33, 34]. P62 is another autophagy-related protein, which is selectively incorporated into autophagosomes through direct binding to LC3-II and is efficiently degraded in the autophagy [35]. We measured the signature proteins involved in autophagy, such as Beclin-1, LC3, and P62. The results of qRT-PCR, western blotting, and IHC showed that the upregulation of Beclin-1 and LC3 could be reversed by fenofibrate pretreatment. This was also the case for P62. So, we concluded that fenofibrate could attenuate autophagy during IR.

Next, we need to explore the mechanisms of fenofibrate on how to attenuate the injury of IR. PPAR-*α* is a key transcription factor that mediates the nucleus–mitochondrial interactions to regulate inflammation, lipid metabolism, and mitochondrial functions in various tissues and cells, and it has become a main target in NFALD [36, 37]. PPAR-*α* activation increases the expression of sirtuin-1 (SIRT1), which inhibits NF-*κ*B, depending on the AMPK pathway and thus reducing inflammation [38, 39]. Dealing with hyperlipidemia, fenofibrate decreased TNF*α* and IFN-*γ*, but the levels had increased in PPAR*α* knockout mice [40]. It was reported that PPAR-*α* activation could decrease IR-induced liver, heart, and brain injury by suppressing inflammation, apoptosis, and lipid peroxidation [41, 42]. Furthermore, PPAR-*α* can promote the expression of Bcl-2 and subsequently inactivate apoptosis [43]. An experimental study suggested that fenofibrate could inhibit apoptosis through SIRT1-mediated deacetylation of FoxO1 [44]. PPAR-*α* can also regulate hepatocyte autophagy. Evidence showed that PPAR-*α* activation attenuated the immune response and protect liver from acute failure through autophagic activation [45]. A study reported that SIRT3, as the upstream of PPAR-*α*, can regulate the expression of PPAR*α* to affect autophagy [46]. However, the role of FF in mouse IR injury was largely unknown. Therefore, we need to determine the expression of PPAR-*α* to assess whether it also took part in IR injury and found that PPAR-*α* expression was upregulated in fenofibrate preconditioning groups.

AMPK is a serine/threonine kinase that can be activated by ATP depletion, antioxidant interference, and ROS production [47]. In hepatocytes, AMPK activation is necessary for response to diverse metabolic stresses like inflammation, hypoxia, and oxidative stress [48]. Moreover, activating the AMPK pathway could attenuate oxidative stress and exhibit further protective effects through preventing ROS production [49]. There are plenty of evidence showing that via the AMPK signaling pathway, acute liver injury, nonalcoholic fatty liver diseases, and even hepatocellular carcinoma can be attenuated [50–53]. Also, Padrissa-Altes et al. found that the upregulation of AMPK activity acted in hepatic cold ischemia and reperfusion [54]. AMPK is known to suppress inflammation, through reducing proinflammatory marker and NF-*κ*B levels [55]. In addition, AMPK activation can ultimately contribute to the inhibition of apoptosis via inhibiting proinflammatory cytokines or ROS production. AMPK signaling activation promoted autophagy in hepatocyte, which related to the mTOR signal transduction pathway to control autophagic proteolysis [32]. As mentioned above, AMPK is associated with ROS and can also stimulate nuclear accumulation of Nrf2 [56]. Nrf2, which is an essential transcription factor for modulating the intracellular adaptive antioxidant response to oxidative stress. It was regarded to be vital for defending against oxidative stress and inflammation through antioxidant cascades [57]. Accumulating evidence from studies indicates that the Nrf2/HO-1 pathway is closely involved in alleviating hepatic I/R injury [58, 59]. We indeed observed that Nrf2 expression and accumulation were enhanced in the fenofibrate treatment groups, in which AMPK was phosphorylated. Nevertheless, the detailed mechanism of Nrf-2 remains to be determined by searching for downstream or upstream gene proteins. In our study, we just elucidate its relevance to HIR in brief.

It has been reported that AMPK, the activation of which is a trigger to downstream mediators such as SIRT-1, PGC-1*α*, and PPAR-*α*, helps modulate hepatic lipid metabolism for the improvement of liver injury [60]. It is well-documented that phosphorylated AMPK (p-AMPK) can activate PPAR*α* [61]. AdipoRon, a drug for restoration of DKD, performed protective role against lipotoxicity and oxidative stress by enhancing the AMPK/PPAR*α* pathway [62]. Liu et al. clarified that autophagy induction in a D-GalN/LPS model appeared to form a hepatoprotective mechanism that may involve AMPK and PPAR*α* [52]. Furthermore, AICAR, an activator of AMPK, enhanced PPAR-*α* expression by promoting PPAR-*α* transcriptional activity, while treating with compound C, an AMPK inhibitor, could reverse the expression of PPAR-*α*, which illustrated that PPAR-*α* activation was in an AMPK-dependent way [52, 61, 63]. Results showed that compared with the IR group, total AMPK stayed unchanged while p-AMPK was significantly upregulated in the FF group, in which the expression of PPAR-*α* was increased. In the meantime, the expression of apoptosis- and autophagy-related proteins was consistent with our idea. We concluded that fenofibrate protected the liver from damage by activating the AMPK/PPAR-*α* pathway and accordingly inhibited apoptosis and autophagy.

Thus, our study showed that fenofibrate can activate PPAR-*α* via the phosphorylation of AMPK, which could alleviate apoptosis and autophagy in hepatic IR injury 6). However, there are several limitations to our study. Fenofibrate and its exact targets and pathway in HIR still require further investigation. Lots of trials need be conducted to verify whether fenofibrate can effectively protect the liver from IR injury clinically.

## 5. Conclusion

Taken together, fenofibrate alleviated hepatic ischemia/reperfusion injury in mice effectively. Fenofibrate reduced the release of inflammatory cytokines and inhibited hepatocyte apoptosis, autophagy, and ROS. And this protective effect is partly based on the activation of the AMPK/PPAR-*α* pathway.

## Figures and Tables

**Figure 6:**
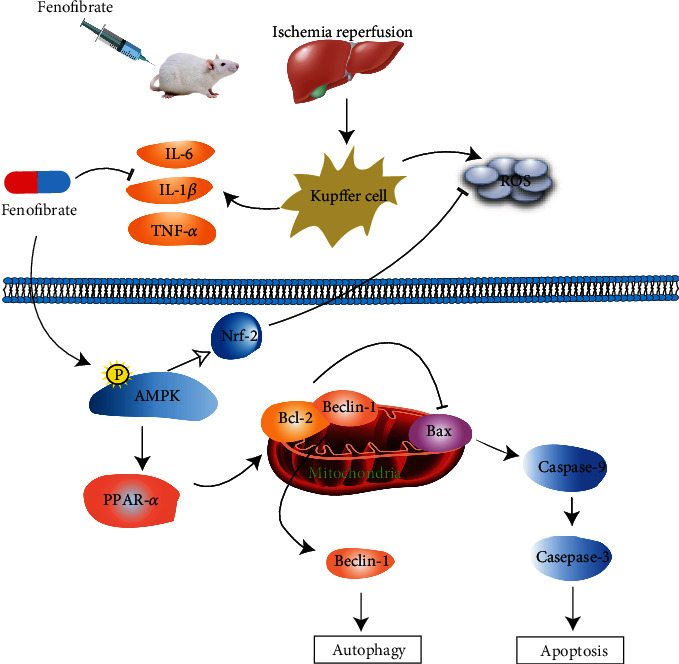
Possible mechanisms of fenofibrate during hepatic IR injury. In our IR-induced liver injury model, activated Kupffer cells promoted the release of proinflammatory cytokines such as TNF-α, IL-1*β*, and IL-6 and production of ROS. Fenofibrate can activate PPAR-*α* via phosphorylating AMPK, collaboratively regulating inflammation response, apoptosis, and autophagy downstream. Furthermore, AMPK activation increased accumulation of Nrf-2 to reduce ROS generation. FF shows hepatoprotective effects against IR injury by inhibiting inflammation and attenuating apoptosis and autophagy through the AMPK/PPAR-*α* pathway.

**Table 1:** Oligonucleotide sequences of primers used for qRT-PCR.

Gene	DNA strand	Primer sequence (5′-3′)
*β*-Actin	Forward	GGCTGTATTCCCCTCCATCG
	Reverse	CCAGTTGGTAACAATGCCATGT
TNF-*α*	Forward	CAGGCGGTGCCTATGTCTC
	Reverse	CGATCACCCCGAAGTTCAGTAG
IL-1*β*	Forward	GAAATGCCACCTTTTGACAGTG
	Reverse	TGGATGCTCTCATCAGGACAG
Bax	Forward	AGACAGGGGCCTTTTTGCTAC
	Reverse	AATTCGCCGGAGACACTCG
Bcl-2	Forward	GCTACCGTCGTCGTGACTTCGC
	Reverse	CCCCACCGAACTCAAAGAAGG
Casepase-9	Forward	GGCTGTTAAACCCCTAGACCA
	Reverse	TGACGGGTCCAGCTTCACTA
Casepase-3	Forward	CTCGCTCTGGTACGGATGTG
	Reverse	TCCCATAAATGACCCCTTCATCA
Beclin-1	Forward	ATGGAGGGGTCTAAGGCGTC
	Reverse	TGGGCTGTGGTAAGTAATGGA
LC3	Forward	GACCGCTGTAAGGAGGTGC
	Reverse	AGAAGCCGAAGGTTTCTTGGG
P62	Forward	GAGGCACCCCGAAACATGG
	Reverse	ACTTATAGCGAGTTCCCACCA
PPAR-*α*	Forward	AACATCGAGTGTCGAATATGTGG
	Reverse	CCGAATAGTTCGCCGAAAGAA
p-AMPK	Forward	ATTGGATTTCCGAAGTATTGATG
	Reverse	CCTGGTCTTGGAGCTACGTCA

## Data Availability

The data used to support the findings of this study are available within the article.
